# Preventing stigma against the gay community to curb mpox transmission in the Western Pacific region

**DOI:** 10.7189/jogh.14.03012

**Published:** 2024-02-09

**Authors:** Zhenggang Zhu, Xiaoyan Pan

**Affiliations:** 1 School of Nursing, Hunan University of Chinese Medicine, Yuelu District, Changsha, Hunan, China; 2 School of Nursing, Wenzhou Medicine University, Chashan Town, Ouhai District, Wenzhou, Zhejiang, China; 3 School of Health Sciences, Universiti Sains Malaysia, Kubang Kerian, Kelantan, Malaysia

## BACKGROUND

On 14 July 2023, the World Health Organization (WHO) released the mpox (formally known as monkeypox) outbreak report. The Western Pacific region has reported the largest proportion of cases (n = 92 (34%)) in the past three weeks (from 19 June to 10 July 2023) [1]. On 2 August 2023, Thailand reported a total of 120 cases of mpox infection, and about half of the mpox cases reported were among people living with HIV [2]. In Thailand, most mpox cases occurred in large and tourist cities [3]. The rapid increase in mpox cases in Thailand may be related to the full resumption of tourism after the coronavirus disease (COVID-19) and the influx of domestic and international tourists to Bangkok for events such as the large gathering in June for Gay Pride Month. The cumulative number of diagnosed mpox cases in Japan exceeds 100, mostly in men between 20 and 40 years old [4]. On 9 August, the Chinese Center for Disease Control and Prevention released the July 2023 Mpox Epidemic Surveillance Report, which showed that there were 491 new confirmed cases of mpox in mainland China, a significant increase from 106 new cases in June. All the new cases were male, and 96.3% were clearly among men who have sex with men (MSM). Most close contacts other than sexual contacts were not infected [5].

## PROBLEMS RELATED TO MPOX TRANSMISSION IN THE WESTERN PACIFIC REGION

The spread of the mpox epidemic in the gay community may fuel the stigma of this highly marginalised community [6]. Reports of mpox cases from WHO and other countries have brought the gay community into the public spotlight. Mpox is more common in the gay community with MSM rather than in homosexuals, such as lesbians and bisexuals, based on previous reports. The issue of MSM transmission leading to mpox has sparked heated discussions. The gay community is still a sensitive topic in the Western Pacific region, which is dominated by developing and less developed countries, and most countries and regions do not accept this particular group. The public lacks sufficient understanding of the gay community, and there even exists the belief that the gay community is immoral [7]. The high-risk group for mpox is the gay community, allowing people to mislabel mpox as a promiscuous, gay disease [8–10]. Several reports of mpox patients with severe symptoms and even death, often with HIV and/or syphilis, have added to the public panic [11,12].

The close link between gays, HIV, sexually transmitted diseases, and mpox has led to stigmatisation and discrimination against the gay community. Some individuals blame and shame the gay community, suggesting they ‘introduced’ the infection into new populations and deserved the infection due to their sexual practices [12]. Misrepresentation in the media can potentially reinforce discriminatory stereotypes of mpox as a gay disease. The gay community faced increased stigma and discrimination, which was even more dangerous than the spread of the mpox virus.

## WHY IS THERE A STIGMA AGAINST THE GAY COMMUNITY DURING MPOX EPIDEMICS

To date, the reported cases of mpox globally have been concentrated in the gay community. The gay community, at risk of mpox, primarily experiences transmission through sexual behaviour. Of the information available about sexual orientation, 87.3% were gay, bisexual, and other people who have sex with men. Of all reported types of transmission, skin and mucous membrane contact during sexual behaviour was the most common, accounting for 71.7% of all transmission events. The most common environment for infection was a party setting with sexual contact, representing 43.4% of all exposures [13]. It is worth noting that the gay community, an at-risk population of mpox, also exhibits a high prevalence of HIV, which can be transmitted through sexual contact within the community. There is a concern that individuals infected with mpox may also be carriers of HIV. People with HIV, being relatively immune-compromised, may face an increased risk of severe illness and death if infected with mpox. Several reports from the USA and Europe suggest that around 40% and up to 90% of cases in some settings occurred in people with HIV [14,15]. In a recent study in Nigeria, HIV-infected patients were at higher risk of mpox infection and death from the disease [16]. As the disease prolonged, lesions were larger secondary to bacterial skin infections and genital ulcers in hospitalised patients [17]. In the USA, the majority of severely ill patients with mpox are blacks with AIDS and untreated HIV infection [18].

## THE HARM OF STIGMA AGAINST THE GAY COMMUNITY

One of the main challenges facing the gay community remains their social acceptance [19]. Reports of mpox against the gay community have further reduced their acceptability [20]. Individuals experiencing stigmatised identities feel vulnerability and discrimination [9]. In addition, infected people are labelled as gay regardless of their sexual orientation. The stigma associated with mpox may adversely affect the prevention and treatment. Stigmatising the gay community increases the risk of spreading mpox, and infected people are less likely to disclose symptoms or seek care, even hide their condition for fear of being diagnosed, thus might lead to delayed access to care. Undiagnosed and untreated mpox infections may infect others through unprotected sex [21]. Stigma could hinder engaging patients in proper care and getting honest answers during contact tracing [22]. Because of the fear of revealing personal privacy, the transmission of mpox among MSM is insidious, the source of infection is unclear, and prevention and control are difficult. Stigmatising or discriminating against mpox patients, which will not help and will certainly cause more harm, can be as dangerous as the mpox virus. It may pose an obstacle to preventing and controlling mpox, indirectly contributing to outbreaks.

## WHY DEFINING MPOX AS A GAY DISEASE IS WRONG

No race, group or animal should be discriminated against in the mpox epidemic. Therefore, the WHO has recently renamed monkeypox as mpox. Although the gay community is the most at-risk of mpox, it should not be stigmatised and discriminated against by wrongly labelling mpox as a ‘gay disease’. In fact, over the past 50 years, the age distribution of mpox infections in Africa revealed that the susceptible population to mpox consisted mainly of children with weakened immune systems, not gay groups [23]. Similarly, mpox is not only transmitted sexually but also through prolonged face-to-face respiratory droplets or through direct or indirect contact with a patient’s body fluids, diseased tissues, and possibly through contaminated pollutants such as clothing and bed linen stained with fluids from ruptured patient’s rashes, and in addition, mpox can be transmitted vertically from mother to child [24]. Therefore, simply defining mpox as gay disease and the transmission mode as sexual transmission is incomplete and may mislead people’s perception of mpox, which is a barrier to preventing the disease or eliminating discrimination.

## THE POSSIBLE PATHWAYS FOR IMPROVEMENT TO PREVENT STIGMA AGAINST THE GAY COMMUNITY

In a correspondence published in April 2023, Irshad, a gay, minoritised trainee physician and mpox patient, used his own experience to demonstrate the importance of inclusion, acceptance, and embracing in overcoming the stigma of mpox [25]. To raise public awareness, eliminate the stigma of mpox and prevent its spread, we suggest the following recommendations to the government and health care sectors.

The publicity of mpox in the mainstream media should be strengthened to ensure the information is authoritative and correct, correcting any existing cognitive biases. It should be emphasised that mpox can affect anyone regardless of gender, age, or sexual orientation, not just the gay community. Moreover, it should be emphasised that transmission routes include respiratory droplets or direct or indirect contact with patient fluids, diseased tissue, and possibly contaminated pollutants, not just sexual transmission.

Strict rules must be established to avoid using emotionally charged language, inaccurate content, fake news and sensationalised headlines in the media to gain social media traffic. Also, disciplinary measures should be imposed on organisations and individuals who broadcast false information or make discriminatory statements against the gay community.

Further, correct publicity strategies should be developed to guide the public to correctly understand mpox, protect the privacy of the gay community, and avoid excessive highlighting of the gay community, which leads to anxiety of the gay community and the public’s fear of the gay community. It must be emphasised the gay community should not be responsible for the spread of mpox. They should not be condemned; instead, they are the main victims and should be given more attention, understanding and care.

Moreover, mpox prevention should be integrated with HIV control efforts to develop interventions for high-risk groups. Additionally, health education on mpox prevention should be provided concurrently with HIV testing. Social welfare organisations should be mobilised, and peer education should be employed to deliver health education to MSM in locations frequented by the gay community, such as bars, parties, and bathrooms. Awareness among MSM and the HIV population regarding protection against mpox infection should be increased, the avoidance of unprotected sex should be encouraged, and advice should be given for consulting a doctor in case of suspected pox-related symptoms.

Equal and non-discriminatory dialogues with the gay community should be encouraged, and open communication between representatives of the gay community, public health experts, researchers, and policymakers should be promoted. It is important to understand the needs of the gay community and develop education and training programs focusing on their sexual health. Moreover, guidance on self-health monitoring for high-risk groups should be promoted.

Finally, the development of the mpox vaccine should be accelerated to improve its effectiveness and reduce side effects. Further, it is important to understand the willingness of the gay community, including gays, bisexuals, and HIV carriers, to be vaccinated against mpox and expand the scope for high-risk groups.

## CONCLUSIONS

Currently, mpox cases are increasing significantly in the Western Pacific region and show no signs of slowing down. Due to the prevalence of mpox, public stigmatisation and discrimination against the gay community have intensified, becoming a complex social issue rather than just a public health issue. We call on public health experts and policymakers in the Western Pacific region to fully account for the stigma of the mpox outbreak on the gay community and to correct the one-sided, misleading information of the media. We hope that intervening with the measures outlined above can eliminate the stigma and discrimination against the gay community and prevent the further spread of mpox.

## References

[1] World Health Organization. Multi-country outbreak of mpox, external situation report. Available: https://www.who.int/health-topics/monkeypox#tab=tab_1. 2023. Accessed: 21 November 2023.

[2] Thai Newsroom. Thailand has the most mpox cases In southeast Asia. 2023. Available: https://thainewsroom.com/2023/08/02/thailand-has-the-most-mpox-cases-in-southeast-asia/. Accessed: 15 October 2023.

[3] Bangkok Post. Bangkok the worst hit by monkeypox virus' spread. 2023. Available: https://www.bangkokpost.com/thailand/general/2633254/bangkok-the-worst-hit-by-monkeypox-virus-spread. Accessed: 28 October 2023.

[4] Outbreak News Today. Japan monkeypox cases top 100. 2023. Available: https://outbreaknewstoday.com/japan-monkeypox-cases-top-100/. Accessed: 20 December 2023.

[5] Chinese Center for Disease Control and Prevention. Monkeypox Outbreak Surveillance for July 2023. 2023. Available: https://www.chinacdc.cn/jkzt/crb/qt/szkb_13037/gwjszl_13092/202308/t20230809_268502.html. Accessed: 22 December 2023.

[6] Bragazzi NL Khamisy-Farah R Tsigalou C Mahroum N Converti M Attaching a stigma to the LGBTQI+ community should be avoided during the monkeypox epidemic. J Med Virol. 2023;95:e27913. 10.1002/jmv.27913 35655436

[7] Cui Y, Yamashita N, Liu M, Lee YC. So Close, yet So Far: Exploring Sexual-minority Women's Relationship-building via Online Dating in China. Proceedings of the 2022 CHI Conference on Human Factors in Computing Systems; 2022 Apr 29–May 5; New Orleans, USA. New York: Association for Computing Machinery; 2022.

[8] Irshad U Overcoming stigma-contracting mpox as a minoritised trainee. Lancet Infect Dis. 2023;23:400. 10.1016/S1473-3099(23)00064-6 36828003

[9] Banjar WM Alaqeel MK Monkeypox stigma and risk communication; Understanding the dilemma. J Infect Public Health. 2023;S1876–0341(23)00068-0. 10.1016/j.jiph.2023.03.002 37002063 PMC10014508

[10] Nimbi FM Baiocco R Giovanardi G Tanzilli A Lingiardi V Who Is Afraid of Monkeypox? Analysis of Psychosocial Factors Associated with the First Reactions of Fear of Monkeypox in the Italian Population. Behav Sci (Basel). 2023;13:235. 10.3390/bs13030235 36975260 PMC10045762

[11] Bui IT Sloan B Tribble M Moore AY An HIV-positive man with painless ulcer and pustules: mpox, syphilis, or both? Proc Bayl Univ Med Cent. 2023;36:510–3. 10.1080/08998280.2023.2193130 37334094 PMC10269416

[12] Hoffmann C Jessen H Wyen C Grunwald S Noe S Teichmann J Clinical characteristics of monkeypox virus infections among men with and without HIV: A large outbreak cohort in Germany. HIV Med. 2023;24:389–97. 10.1111/hiv.13378 36059149

[13] World Health Organization. Multi-country outbreak of monkeypox, external situation report. Geneva: World Health Organization; 2022. Available: https://www.who.int/publications/m/item/multi-country-outbreak-of-monkeypox–external-situation-report–9—2-november-2022. Accessed: 20 November 2023.

[14] Mitjà O Alemany A Marks M Lezama Mora JI Rodríguez-Aldama JC Torres Silva MS Mpox in people with advanced HIV infection: a global case series. Lancet. 2023;401:939–49. 10.1016/S0140-6736(23)00273-8 36828001

[15] Thornhill JP Barkati S Walmsley S Rockstroh J Antinori A Harrison LB Monkeypox Virus Infection in Humans across 16 Countries - April-June 2022. N Engl J Med. 2022;387:679–91. 10.1056/NEJMoa2207323 35866746

[16] Yinka-Ogunleye A Dalhat M Akinpelu A Aruna O Garba F Ahmad A Mpox (monkeypox) risk and mortality associated with HIV infection: a national case-control study in Nigeria. BMJ Glob Health. 2023;8:e013126. 38035733 10.1136/bmjgh-2023-013126 PMC10689363

[17] Ogoina D Iroezindu M James HI Oladokun R Yinka-Ogunleye A Wakama P Clinical Course and Outcome of Human Monkeypox in Nigeria. Clin Infect Dis. 2020;71:e210–14. 10.1093/cid/ciaa143 32052029

[18] Miller MJ Cash-Goldwasser S Marx GE Schrodt CA Kimball A Padgett K Severe Monkeypox in Hospitalized Patients - United States, August 10-October 10, 2022. MMWR Morb Mortal Wkly Rep. 2022;71:1412–7. 10.15585/mmwr.mm7144e1 36327164 PMC9639440

[19] Singla RK Singla S Shen B Biased studies and sampling from LGBTQ communities created a next-level social stigma in monkeypox: a public health emergency of international concern (PHEIC). Indo Glob J Pharm Sci. 2022;12:205–8. 10.35652/IGJPS.2022.12025

[20] Dsouza VS Rajkhowa P Mallya BR Raksha DS Mrinalini V Cauvery K A sentiment and content analysis of tweets on monkeypox stigma among the LGBTQ+ community: A cue to risk communication plan. Dialogues Health. 2023;2:100095. 10.1016/j.dialog.2022.100095 36573228 PMC9767808

[21] Kowalski J Cielniak I Garbacz-Łagożna E Cholewińska-Szymańska G Parczewski M Comparison of clinical course of Mpox among HIV-negative and HIV-positive patients: A 2022 cohort of hospitalised patients in Central Europe. J Med Virol. 2023;95:e29172. 10.1002/jmv.29172 37861345

[22] Baldovin T Girolametto G Geppini R Bordignon M Alaibac M Preventing and fighting stigma: a lesson from the first Mpox in Veneto region of Northeast Italy-A case report. Front Public Health. 2023;11:1141742. 10.3389/fpubh.2023.1141742 37275485 PMC10235756

[23] Yao KH Learning from the past: the history of human monkeypox and the atypical multi-country outbreak in 2022. Zhongguo Dang Dai Er Ke Za Zhi. 2022;24:717–27. 35894184 10.7499/j.issn.1008-8830.2206019 PMC9336622

[24] Kaler J Hussain A Flores G Kheiri S Desrosiers D Monkeypox: A Comprehensive Review of Transmission, Pathogenesis, and Manifestation. Cureus. 2022;14:e26531. 10.7759/cureus.26531 35928395 PMC9345383

[25] Irshad U Overcoming stigma-contracting mpox as a minoritised trainee. Lancet Infect Dis. 2023;23:400. 10.1016/S1473-3099(23)00064-6 36828003

